# Increase in invasive disease caused by *Haemophilus influenzae* b, the Netherlands, 2020 to 2021

**DOI:** 10.2807/1560-7917.ES.2021.26.42.2100956

**Published:** 2021-10-21

**Authors:** Anneke Steens, Kamelia R Stanoeva, Mirjam J Knol, Rob Mariman, Hester E de Melker, Nina M van Sorge

**Affiliations:** 1 Centre for Infectious Disease Control, National Institute for Public Health and the Environment (RIVM), Bilthoven, the Netherlands; 2 European Public Health Microbiology Training Programme (EUPHEM), European Centre for Disease Prevention and Control (ECDC), Stockholm, Sweden; 3 Department of Medical Microbiology and Infection Prevention, Amsterdam UMC, location AMC, University of Amsterdam, Amsterdam, the Netherlands; 4 Netherlands Reference Laboratory for Bacterial Meningitis, Amsterdam UMC, location AMC, University of Amsterdam, Amsterdam, the Netherlands

**Keywords:** *Haemophilus influenzae* type b, incidence, hexavalent vaccine, vaccination, National immunisation programme

## Abstract

The incidence of most respiratory-transmitted diseases decreased during the COVID-19 pandemic as a result of containment measures. In contrast, in the Netherlands we noted an increase in invasive disease caused by *Haemophilus influenzae* b (Hib) (from < 0.3/100,000 before 2019 to 0.39 and 0.33/100,000 in 2020 and 2021) in vaccinated and unvaccinated age groups. We did not find a change in vaccine effectiveness against Hib invasive disease (effectiveness > 90%). We discuss factors that may have contributed to this rise.

*Haemophilus influenzae* serotype b (Hib) is a vaccine-preventable disease, which has had a fluctuating but low incidence as a result of childhood vaccination. However, we noted an unexpected increase in the Netherlands in 2020 and 2021 in vaccinated and non-vaccinated age groups. As the increase occurred during the COVID-19 pandemic when several other respiratory transmitted diseases decreased, the change in incidence was especially unexpected. The aim of this study was to describe and provide possible explanations for the observed unexpected increase in Hib disease incidence compared to the previous decades.

## Increase in *Haemophilus influenzae* b invasive disease

**Figure 1:**
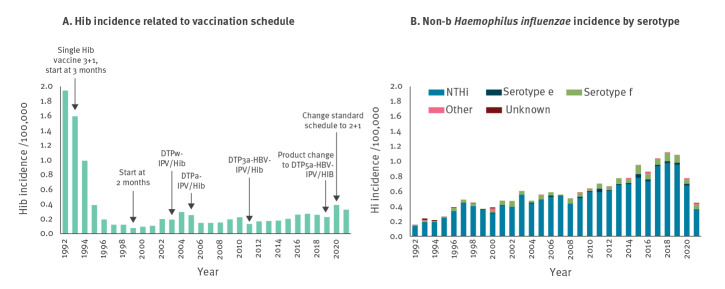
Incidence of invasive disease caused by *Haemophilus influenzae* serotype b in relation to changes in the childhood immunisation programme (A) and disease incidence caused by non-typeable and non-b *H. influenzae* serotypes (B), the Netherlands, 1992–2021^a^

**Figure 2:**
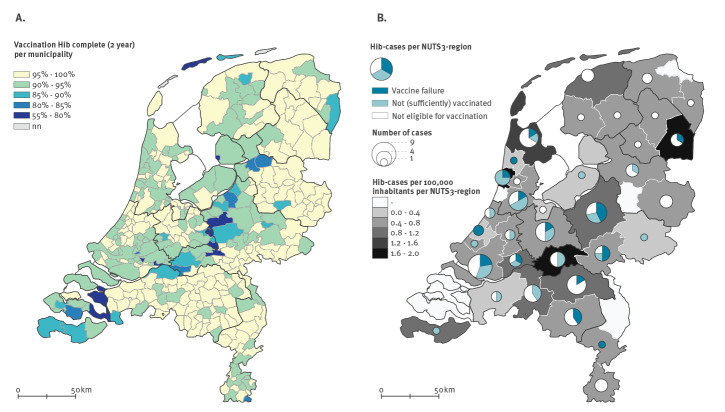
*Haemophilus influenzae* serotype b (Hib) vaccine coverage at municipal level in children born in 2018 (A), Hib incidence at NUTS3-level and geographical distribution of Hib cases (n = 103) by vaccine status (B), the Netherlands, January 2020–August 2021

**Figure 3:**
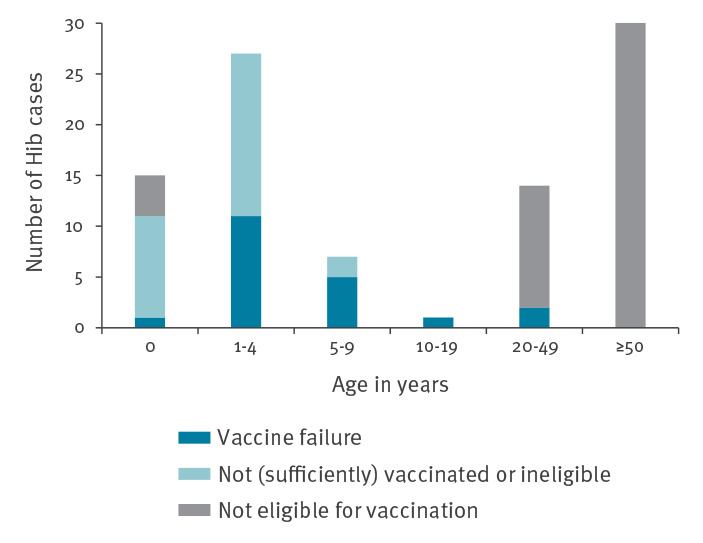
Age distribution of *Haemophilus influenzae* serotype b cases, by vaccination status, the Netherlands, January 2020–August 2021 (n = 105)

Vaccination against *Haemophilus influenzae* serotype b (Hib) was introduced in the childhood immunisation programme of the Netherlands in 1993. After a prompt decrease, the annual Hib incidence fluctuated but remained below 0.3 cases per 100,000 during the period 1996 to 2019 (Figure 1 panel A). In 2020 and 2021 (extrapolated for 2021, data up to and including August), the overall incidences were 0.39 and 0.33 per 100,000, respectively. No clear geographical clustering was observed (Figure 2 panel B). Forty per cent of cases occurred in children under the age of 5 years (Figure 3). The incidence in this age group was below 2.6 per 100,000 between 1996 and 2019, although an increasing trend has been observed since 2012 from 0.5 to 1.0 per 100,000 to 2.0 per 100,000 in 2019. In 2020 and 2021 respectively, the incidence in the age group under 5 years was 3.3 per 100,000 (n = 28) and 2.6 per 100,000 (n = 15 within the first 8 months). Although secular trends occur for Hib, the current increase stands out because it coincides with the coronavirus disease (COVID-19) pandemic, during which a decrease in several other respiratory invasive diseases was observed in many countries [1,2]. Correspondingly, the incidence of non-type b *H. influenzae* invasive disease in the Netherlands decreased by 40% during 2020 and 2021 compared with 2015 to 2019 (Figure 1 panel B). These decreases were probably caused by reduced transmission resulting from COVID-19 containment measures, but changes in referral and testing policy cannot be excluded [3].

DTPa-IPV/Hib: vaccination against diphtheria, tetanus, pertussis (acellular, component), polio (inactivated) and Hib; DTPw-IPV/Hib: vaccination against diphtheria, tetanus, pertussis (whole cell), polio and Hib; DTP5/3a-HBV-IPV/Hib: vaccination against diphtheria, tetanus, pertussis (acellular, component), hepatitis B, polio (inactivated) and Hib; Hib: *Haemophilus influenzae* serotype b; NTHi: non-typeable *Haemophilus influenzae*.

^a^ Data for 2021 are extrapolated based on data up to and including August.

Hib: *Haemophilus influenzae* serotype b; nn: nomen nescio; NUTS: nomenclature of territorial units for statistics.

Panel A shows the vaccine coverage at 2 years of age in 2020, i.e. the percentage of fully vaccinated children born in 2018. For three cases, the postal code was unavailable and they are therefore not presented here. Here, nn indicates that the incidence cannot be shown because of privacy reasons as the municipality is too small to keep confidentiality.

Hib: *Haemophilus influenzae* serotype b.

Seventeen of the 19 vaccinated cases had received four doses. Note that one case in the age group 1-4 years had unknown vaccine status and is therefore not presented in this figure.

## Changes in the vaccination schedule

Changes related to Hib vaccination in the childhood immunisation programme (Figure 1 panel A) included a change from the pentavalent to a hexavalent composition (DT3aP-HBV/Hib) in 2011 and subsequently a product change to DT5aP-HBV/Hib in 2019. DT3aP-HBV/Hib and DT5aP-HBV/Hib differ in composition of the carrier compound, adjuvant and length of the Hib component [4]. Finally, from 2020, the 3+1 schedule with primary doses at the age of 2, 3 and 4 month and a booster dose at 11 months changed to a 2+1 schedule (3, 5 and 11 months). Children of mothers who have not received maternal vaccination (ca 30%) or who are born prematurely, follow a 3+1 schedule with vaccination at 2, 3, 5 and 11 months.

## Vaccine failure and effectiveness

Vaccine failure was defined for those younger than 12 months as a Hib case who had received at least two doses of Hib-containing vaccine. For those 1 year and older, the primary series and a booster at 11 months or at least one dose given after the first birthday counted as vaccine failure. Only doses given more than 14 days before disease onset counted as vaccination. 

**Figure 4:**
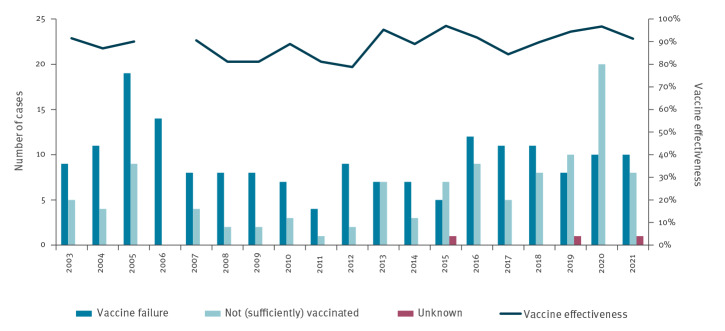
Number of *Haemophilus influenzae* serotype b cases in individuals older than 3 months and born in or after April 1993, by vaccine status, and estimated vaccine effectiveness (screening method), the Netherlands, 2003 to 2021 (n = 290)

In 2015 to 2019, on average 9.4 vaccine failures occurred annually (55% of cases in the vaccine-eligible population, i.e. born in or after April 1993 and older than 3 months). Ten vaccine failures occurred in 2020 and another 10 have so far occurred in 2021 by the end of August (Figure 4). The number of cases in not (sufficiently) vaccinated individuals increased twofold in 2020 (n = 20) compared with the previous 5 years (on average n = 8) and numbers for the first 8 months of 2021 have already reached the 5-year average (Figure 4). Based on the screening method [5] using the population vaccine coverage measured in 2-year-olds [6], the vaccine effectiveness (VE) was estimated at 97% (95% confidence interval (CI): 93–99) in 2020 and 91% (95% CI: 78–97) in 2021 compared with 92% (95% CI: 88–95) in 2015 to 2019. Overall, the VE therefore seems unchanged.

The numbers for 2021 only include data until and including August. It is expected that the number for the entire year will still increase. In 2006, all cases were sufficiently vaccinated, i.e. vaccine failures; therefore, vaccine effectiveness could not be estimated.

Note that, based on data of the first measles-mumps-rubella (MMR) vaccination [1], some delay in vaccination during the start of the COVID-19 pandemic can also be expected for Hib. However, after catch-up vaccination, participation in the first MMR vaccination only lags 1–2% behind compared with the year before.

### Increase in cases seen both in and outside the Bible Belt

**Figure 5:**
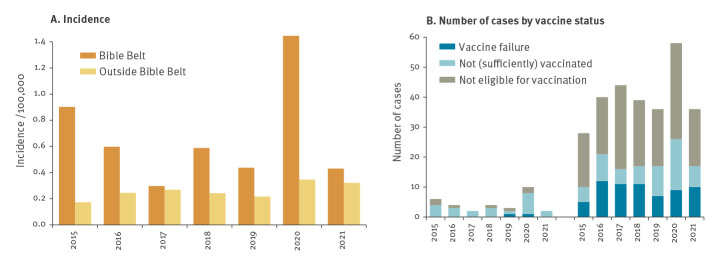
*Haemophilus influenzae* serotype b occurrence in and outside the Bible Belt, the Netherlands, 2015 to 2021 (n = 312)

Although the vaccine coverage for Hib at age 2 years is high in the Netherlands with 93% [6], there are clustered religious municipalities where vaccine coverage is substantially lower, the so-called Bible Belt (Figure 2 panel A) [7]. We compared the Hib incidence in these municipalities with the rest of the country. Bible Belt communities were selected based on an MMR vaccine coverage lower than 90% during the period 2015 to 2021. Overall, 9.3% of the cases (n = 31) in the period 2015 to 2021 resided in Bible Belt municipalities. The Hib incidence was especially high in the Bible Belt in 2020 (1.45/100,000; Figure 5) but the incidence outside the Bible Belt was also higher in 2020 and 2021 (0.35 and 0.32/100,000, respectively, compared with 0.17 and 0.27/100,000 in 2015–2019).

The incidence of 2021 is extrapolated from the first 8 months of the year. Dotted line: average incidence in the Netherlands during 2015–2019.

## Ethical statement

In accordance with Dutch law, approval from a medical ethics committee was not deemed necessary since cases were not subject to any actions or rules of conduct. Data regarding cases were obtained by use of standard surveillance procedures, and pseudonymised data were used in the study. Informed consent was not obtained, as the collection of data complies with the exceptions for not asking informed consent as formulated in the Dutch Implementation Act General Data Protection Regulation.

## Discussion

The increase in Hib incidence may be explained by methodological, behavioural, biological or vaccine-related factors. Changes in surveillance method could affect the detected number of cases. In the Netherlands, all *H. influenzae* isolated from normally sterile sites are submitted for serotyping to the National Reference Laboratory for Bacterial Meningitis (NRLBM) on a voluntary basis. In addition, clinicians or laboratories should notify Hib cases to the municipal health centres. Laboratory and clinical data are merged for surveillance by the National Institute for Public Health (RIVM). This has been the policy for many years. Focused communication on the importance of notification took place in March 2019 [8]. Possible effects of this communication would probably affect the incidence of all *H. influenzae* serotypes as serotyping is only performed by the NRLBM. However, in contrast to the Hib incidence, the incidences of non-b *H. influenzae* serotypes were substantially lower in 2020 and 2021 compared with previous years, despite an increase in non-typeable *H. influenzae* before the COVID-19 pandemic.

The pandemic has changed the pattern of interaction in the society [9]. As suggested by the association between the incidence of COVID-19 hospitalisation and religion [10], the changes in inter-personal interactions and possibly adherence to guidelines may have been different in the Bible Belt vs the rest of the country. Such differences may explain the high percentage of unvaccinated cases in 2020 but not the increase in incidence in and outside the Bible Belt.

The increase in Hib in vaccinated and unvaccinated age groups may be explained by increased transmission, e.g. because of increased colonisation or by increased invasiveness. Generally, Hib vaccination in childhood immunisation programmes has resulted in very low Hib colonisation rates [11,12]. Our recent data show a shift in the incidence of subcluster A within the most prevalent sequence type (ST-6) among invasive Hib isolates [13]. The subclusters were determined using principle component analysis on core genome multilocus sequence typing of 80 isolates from 1986 to 2018. Preliminary data reveal a further expansion of this subcluster in 2019 to 2021 (data not shown). Whether this dominant clone is a more efficient coloniser or has more invasive traits is currently being investigated. We are planning to study whether the prevalence of asymptomatic Hib colonisation and ST-6 subcluster A colonisation in children has changed over time. Although preliminary, this clone seems not to be a vaccine escape variant (data not shown).

Overall, the data presented here do not indicate a change in Hib VE. However, immunogenicity studies show lower systemic titres of IgG anti-polyribosylribitol phosphate after booster vaccination with DT5aP-HBV/Hib compared with DT3aP-HBV/Hib, for both a 2+1 and a 3+1 schedule [14]. In addition, seroprevalence data from France indicate that a 3+1 immunisation schedule induces a more robust immune response than a 2+1 schedule [15]. Therefore, we are planning to update previous VE analyses [16], including product- and schedule-specific analyses for different times since vaccination.

## Conclusion

Although the absolute number of invasive Hib cases is still low, the increase in incidence is worrisome because of the severity of the disease. It calls for an explanation in order to implement control measures if needed. Furthermore, this underlines the importance of a comprehensive *H. influenzae* surveillance system including data on serotype and vaccination status.
