# Erratum for Euro Surveill. 2021;26(42)

**DOI:** 10.2807/1560-7917.ES.2021.26.43.211028e

**Published:** 2021-10-28

**Authors:** Eurosurveillance editorial team

**Affiliations:** 1 European Centre for Disease Prevention and Control (ECDC), Stockholm, Sweden

In the article ‘Increase in invasive disease caused by Haemophilus influenzae b, the Netherlands, 2020 to 2021’ by Steens et al. published on 21 October 2021, Figure 5 was originally published without the line showing the average incidence in panel A, and without the captions under the x axis in panel B. This was corrected on 28 October 2021. We apologise for the mistake.

